# Integration of Gene Expression Profile Data to Verify Hub Genes of Patients with Stanford A Aortic Dissection

**DOI:** 10.1155/2019/3629751

**Published:** 2019-07-14

**Authors:** Weitie Wang, Tiance Wang, Yong Wang, Hulin Piao, Bo Li, Zhicheng Zhu, Rihao Xu, Dan Li, Kexiang Liu

**Affiliations:** Department of Cardiovascular Surgery, The Second Hospital of Jilin University, Ziqiang Street 218, Changchun, Jilin 130041, China

## Abstract

Thoracic aortic dissection (TAD) is a catastrophic disease worldwide, but the pathogenic genes and pathways are largely unclear. This study aims at integrating two gene expression profile datasets and verifying hub genes and pathways involved in TAD as well as exploring potential molecular mechanisms. We will combine our mRNAs expression profile (6 TAD tissues versus 6 non-TAD tissues) and GSE52093 downloaded from the Gene Expression Omnibus (GEO) database. The two mRNAs expression profiles contained 13 TAD aortic tissues and 11 non-TAD tissues. The two expression profile datasets were integrated and we found out coexpression of differentially expressed genes (DEGs) using bioinformatics methods. The gene ontology and pathway enrichment of DEGs were performed by DAVID and Kyoto Encyclopedia of Genes and Genomes online analyses, respectively. The protein-protein interaction networks of the DEGs were constructed according to the data from the STRING database. Cytohubber calculating result shows the top 10 hub genes with CDC20, AURKA, RFC4, MCM4, TYMS, MCM2, DLGAP5, FANCI, BIRC5, and POLE2. Module analysis revealed that TAD was associated with significant pathways including cell cycle, vascular smooth muscle contraction, and adrenergic signaling in cardiomyocytes. The qRT-PCR result showed that the expression levels of all the hub genes were significantly increased in OA samples (p < 0.05), and these candidate genes could be used as potential diagnostic biomarkers and therapeutic targets of TAD.

## 1. Introduction

Thoracic aortic dissection (TAD) is a catastrophic cardiovascular disease with the separation of the layers of the aortic wall [1]. The traditional treatment of this life-threatening disease includes surgical or hybrid endovascular techniques, but the morbidity and mortality remain unsatisfactory [2]. So necessary understanding of the molecular mechanism may provide new insights into therapeutic targets for TAD. Dysregulated extracellular matrix (ECM) protein like elastin and collagen and depletion of vascular smooth muscle cells (VSMCs) of the aortic wall are believed as the main histopathological findings [3–5]. However, still little is known about the genetics of the disease. 

With the development of the gene expression profile, comparisons of differentially expressed genes that participate in the regulation of pathophysiological conditions in pathological tissue and normal tissue were available and convenient, and mRNAs gene expression profiles have been carried out in many diseases including TAD [6]. Although high-throughput sequencing technologies have provided many diverse expressed genes, different expression profiles in TAD provided various results and no reliable results have been identified up to now [6–8]. The integrated advanced bioinformatics methods may provide a way to solve the disadvantages and identify the hub genes involved in TAD.

In the present study, we use mRNA microarray to acquire differential expression profiles in human TAD tissues and non-TAD tissues. Then we downloaded another microarray dataset GSE52093 [9] and screened out the coexpression of differentially expressed genes (DEGs) between TAD patients and non-TAD patients. Traditional bioinformatics methods including gene ontology (GO) and pathways enrichment analyses of DEGs combined protein-protein interaction (PPI) network and cytohubber calculating software as well as Molecular Complex Detection (MCODE) analysis were used to analyze the key gene and pathway. Thus, these data would provide a foundation for new biomarkers and therapeutic targets for human TAD.

## 2. Materials and Methods

### 2.1. Tissue Collection and Microarray Analysis

This study was conducted in accordance with the Declaration of Helsinki and was approved by the Ethics Committee of the Second Hospital of Jilin University. There are 12 samples (6 TAD and 6 NT) that were used for microarray analysis by Arraystar Human mRNA microarray which contained 14596 mRNAs probes. The differentially expressed mRNAs were statistically significant and the change in threshold values was >2.0- or < −2.0-fold between the two groups and Benjamini-Hochberg corrected P <0.05.

### 2.2. Data Processing and Identification of DEGs

The raw data were preprocessed by affy package [10] in R software and limma package [11] in R software was used to identify the upregulated and downregulated DEGs between our mRNA microarray and GSE52093. P values were adjusted using the Benjamini and Hochberg test, and p < 0.05 and |log⁡FC  | > 1 were considered as the cutoff criterion.

### 2.3. Gene Ontology (GO) and Pathway Enrichment Analyses

DAVID (the Database for Annotation, Visualization, and Integrated Discovery) online bioinformatics database is an analysis tool of biological data to integrate and provide information for biological function and protein list [12]. This tool was used in this study to provide GO enrichment and KEGG (Kyoto Encyclopedia of Genes and Genomes) pathway analysis. GO analysis included categories of cellular component (CC), biological processes (BP), and molecular function (MF). Pathway analysis is a functional analysis that maps genes to KEGG pathways. And gene count >2 and p < 0.05 were set as the cutoff point.

### 2.4. Integration of Protein-Protein Interaction (PPI) Network Analysis

STRING (https://string-db.org/cgi/input.pl) is an online database resource search tool which can provide analysis of interacting genes including physical and functional associations [13]. In this study, the STRING online tool was used to construct a network of upregulation and downregulation differentially expressed genes (DEGs); all the DEGs were input in the Multiple Proteins by Names in STRING online tool. The Basic Settings include meaning of network edges (confidence: line thickness indicates the strength of data support) and active interaction sources (Text mining, Experiments, Databases, Co-expression, Neighborhood, Gene Fusion, Co-occurrence), with a confidence score >0.4 defined as significant. Then the interaction data were typed into the Cytoscape software [14] to structure a PPI network. Based on the above data, we used MCODE [15], a built-in APP in Cytoscape software, to analyze the interaction relationship of the DEGs encoding proteins and screening hub gene. The parameters of MCODE network scoring and cluster finding were set as follows: find clusters = in whole network, degree cutoff = 2, cluster finding = haircut, node score cutoff = 0.2, k-core = 2, and max depth = 100. The parameters of cytoHubba were set as follows: Hubba nodes = top 10 nodes ranked by Degree, display options = check the first-stage nodes, display the shortest path, and display the expanded subnetwork.

### 2.5. Quantitative Reverse Transcription-PCR (qRT-PCR) Validation and Statistical Analysis

qRT-PCR was used to verify the core genes [16]. Total RNA was reverse-transcribed to cDNA using PrimeScript RT reagent Kit with gDNA Eraser (TaKaRa, Japan) according to the manufacturer's instructions. Primer 5.0 software (PREMIER Biosoft, Palo Alto, CA, USA) was used to design primers, and a QuantStudio 7 Flex real-time PCR system (Applied Biosystems, Carlsbad, CA, USA) was used. All primers used in this study are listed in Table 1. All samples were normalized to GAPDH. And the relative expression levels of each gene were calculated using 2−ΔΔCt methods.

## 3. Results

### 3.1. Identification of DEGs in TAD

A total of 13 TAD patients and 11 matched non-TAD patients were analyzed; taking p < 0.05 and FC>2 as a threshold, we extracted 4156 and 2834 DEGs from the expression profile datasets GSE 52093 and our mRNA profile datasets, respectively. By integrated analysis, a total of 433 DEGs were identified, including 281 upregulated DEGs and 152 downregulated DEGs in TAD samples compared with non-TAD samples.

### 3.2. GO Functional Enrichment Analysis

**Figure 1:**
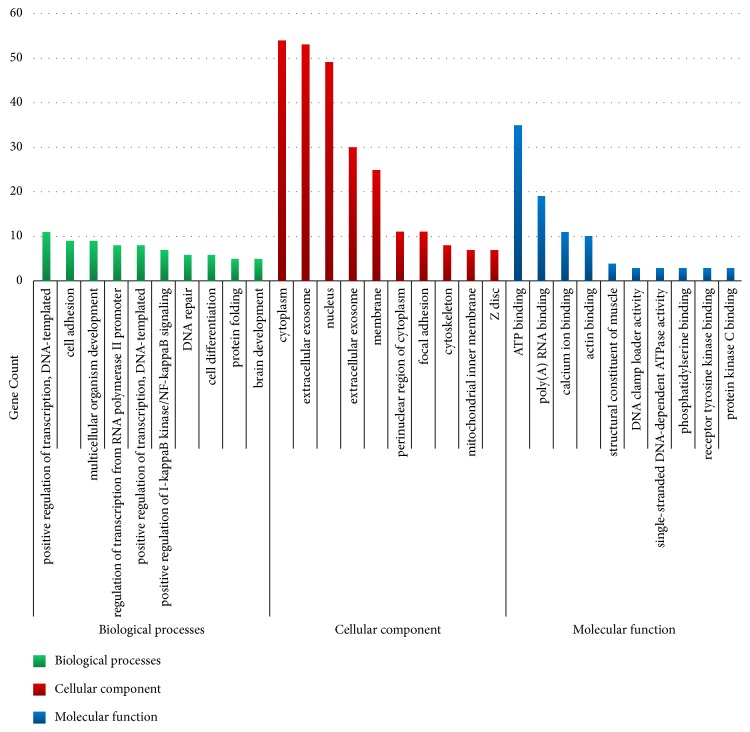
Gene ontology analysis classified the differentially expressed genes into 3 groups: molecular function, biological process, and cellular component.

To know the functions of all DEGs, we used DAVID online tool, and the DEGs functions of GO function enrichment were divided into three groups including BP, CC, and MF (Figure 1). As shown in Figure 1 and Table 2, in the biological processes group, the down-DEGs are mainly enriched in extracellular exosome, cell adhesion, regulation of cell growth, muscle contraction, and collagen fibril organization and the up-DEGs are mainly enriched in positive regulation of I-kappaB kinase/NF-kappaB signaling, DNA replication, DNA repair, DNA replication initiation, and DNA recombination. In the cellular component group, the down-DEGs are mainly enriched in extracellular exosome, focal adhesion, Z disc, actin cytoskeleton, and extracellular matrix and the up-DEGs are mainly enriched in cytoplasm, extracellular exosome, nucleus, membrane, and perinuclear region of cytoplasm. And in the molecular function group, the down-DEGs are mainly enriched in calcium ion binding, actin binding, structural constituent of muscle, phosphatidylserine binding, and receptor tyrosine kinase binding and the up-DEGs are mainly enriched in ATP binding, poly(A) RNA binding, DNA clamp loader activity, single-stranded DNA-dependent ATPase activity, and purine nucleobase binding.

### 3.3. Signaling Pathway Analysis

**Figure 2:**
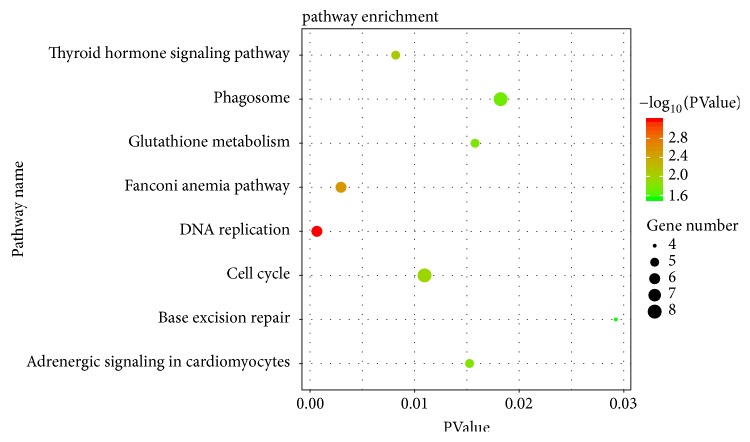
Kyoto Encyclopedia of Genes and Genomes enrichment analysis of the pathways. The gradual color represents the P value; the size of the black spots represents the gene number.

After the pathway enrichment analysis, downregulated genes were mainly enriched in thyroid hormone signaling pathway and adrenergic signaling in cardiomyocytes. And upregulated genes were mainly enriched in DNA replication, fanconi anemia pathway, cell cycle, glutathione metabolism, phagosome, and base excision repair (Figure 2).

### 3.4. PPI Network and Modular Analysis

**Figure 3:**
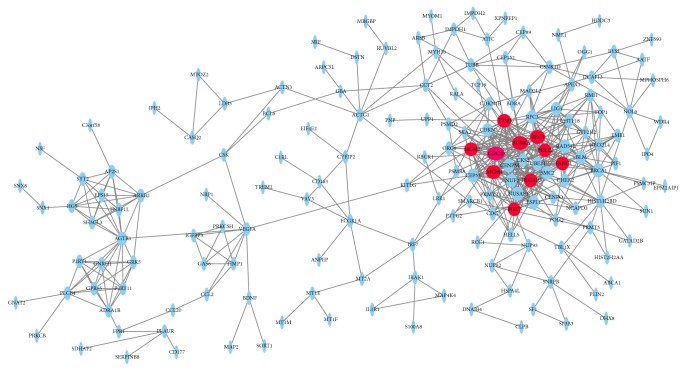
PPI network constructed with the differentially expressed genes. Red nodes represent hub GENE analysis by cytohubba.

**Figure 4:**
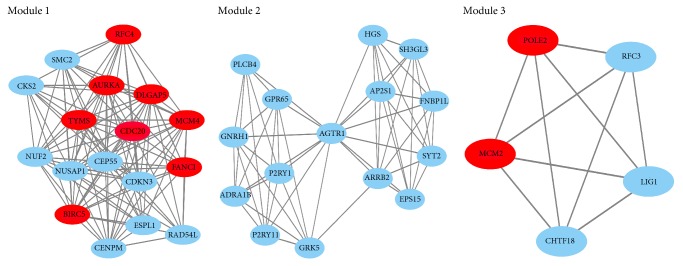
The three most significance modules. Red nodes represent hub GENE analysis by cytohubba.

All the DEGs were analyzed using STRING database. Then we put these data into Cytoscape software to construct a PPI network containing 432 nodes and 379 edges (Figure 3). In these DEGs, 10 hub genes included CDC20, AURKA, RFC4, MCM4, TYMS, MCM2, DLGAP5, FANCI, BIRC5, and POLE2 after calculating. In these 10 hub genes, CDC20 presented with the highest degree (degree = 31). The Cytoscape plugin MCODE shows the top three modules with 14.25, 8.00, and 5.00 scores, respectively, and this is shown in Figure 4. Then the genes in these three modules were performed with functional enrichment analyses. Pathway enrichment analysis showed that Module 1 is mainly relevant with oocyte meiosis, cell cycle, and DNA replication. Module 2 is mainly associated with endocytosis, vascular smooth muscle contraction, adrenergic signaling in cardiomyocytes, cGMP-PKG signaling pathway, and chemokine signaling pathway. Module 3 is mainly associated with DNA replication, nucleotide excision repair, and mismatch repair.

### 3.5. Validation by qRT-PCR of Differentially Expressed mRNAs

**Figure 5:**
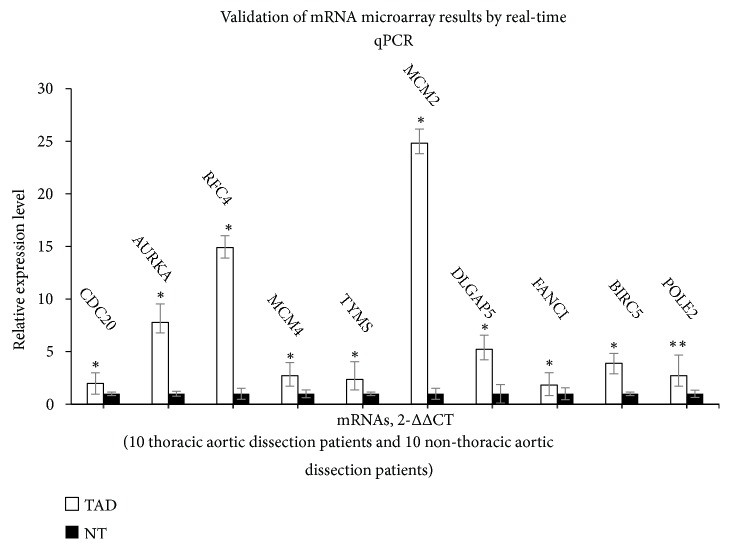
Validation of the top 10 hub genes by qRT-PCR between the TAD group (n = 10) and the non-TAD group (n = 10). All samples were normalized to the expression of GAPDH, and the relative expression levels of each gene were analyzed using the 2−△△Ct method. *∗∗*P < 0.01.

To validate microarray results, the expression levels of top 10 hub genes were determined in ascending aortic samples of thoracic aortic dissection and no thoracic aortic dissection using qRT-PCR. The verification result showed that the expression levels of the 10 hub genes were significantly increased in TAD samples (p < 0.05) (Figure 5). All validations are consistent with the microarray data and analytical results in this study.

## 4. Discussion

TAD is a catastrophic disease of the aorta with an intimal tear that penetrates the aortic media and leads to high mortality. The 3-month mortality rate is approximately 90% in patients without any treatment, and the immediate mortality rate of acute type A aortic dissection increases 1% per hour during the first 48 hours [17–19]. So the fast diagnosis and treatment are necessary. However, the symptoms of TAD are always similar with other diseases, such as pulmonary embolism, acute coronary syndrome, and myocardial infarction and often lead to delay in diagnosis and death [20, 21]. Therefore, making early diagnosis especially noninvasive blood-based test is critical for patients to get timely treatment and survive. Currently, there is no available diagnostic biomarker yet. Thus, the identification of novel and potential biomarkers is particularly important for people with TAD.

Microarray and high-throughput sequencing technologies are powerful tools that can be used to investigate and predict potential targets genes for cardiovascular disease and underlying pathological mechanisms and responses to new treatments. By array analysis, the different expression profiles of diseased and normal tissues can be used to compare systemically and comprehensively. Then bioinformatics analysis can identify the important molecular networks from the expression profiles data. However, most studies focus on a single cohort study and different studies report various results with each other. So this study integrated two cohorts profile datasets from different groups, and bioinformatics methods are applied to analyze the raw data and to identify novel disease mechanisms and new biomarkers with potential clinical applications.

Actually, there are only 12 mRNAs expression profiles in our study which may influence the generalizability of the results. So we choose profile datasets GSE 52093 which contain 7 TAD tissues and 5 non-TAD tissues from GEO database and integrate these two profile datasets which contain 13 TAD tissues versus 11 non-TAD tissues to make the results more generalizable. In this study, we identify 433 DEGs, including 281 upregulated DEGs and 152 downregulated ones. The down-DEGs by GO functional enrichment analysis showed that the downregulated DEGs are mainly enriched in extracellular exosome, cell adhesion, regulation of cell growth, muscle contraction, and collagen fibril organization and the up-DEGs are mainly enriched in positive regulation of I-kappaB kinase/NF-kappaB signaling, DNA replication, DNA repair, DNA replication initiation, and DNA recombination. This conforms to our knowledge that medial degeneration is now thought to be a fundamental mechanism of TAD development and progression. VSMCs are the major cells in aortic media. The function of VSMCs such as proliferation and migration acts critical biomechanical properties of the aortic wall. YAP1 has been reported to mainly exist in aortic media and it was significant downregulation in AD aortic tissue. Further study confirms that Talin-1 regulates VSMC apoptosis and finally causes pathologic vascular remodeling to change vascular media structure and function and lead to TAD [22]. Many genes such as Sirtuin-1, PCSK9, and brahma-related gene 1 had been reported to be associated with the pathophysiologic processes of TAD through influence proliferation and migration of VSMCs [23–25].

KEGG pathways suggested that DNA replication, cell cycle, glutathione metabolism, phagosome, and base excision repair might be involved in TAD development. Like previous high-throughput sequencing studies, we cannot be sure which one is the most relevant mechanism about TAD and the most hub gene about AD is also not certain. So we carry out some further analysis such as PPI network and modular analysis including STRING database, cytohubba analysis, and MCODE analysis which are mainly widely used in inferring the most hub gene and pathways[16]. In our study, the cytohubba analysis shows that the top ten hub genes are CDC20, AURKA, RFC4, MCM4, TYMS, MCM2, DLGAP5, FANCI, BIRC5, and POLE2 after calculating. Actually, there are a lot of methods that can be used to determine whether a gene is a “hub gene.” So we also used CytoNCA which is another tool for calculating network centralities to verify the results calculated by cytohubba. Fortunately, both tools with different algorithms present with the same results. From these hub genes, we infer that the cell cycle may act as an important role in TAD. Then we used Cytoscape plugin MCODE for analysis from another aspect, and the top module included 17 nodes and 114 edges. We choose the nodes for functional enrichment analyses and the cell cycle was the most relevant pathway and also included 8 hub genes which were previously forecasted. So we aim to analyze the highest degree hub gene CDC20 and cell cycle in TAD.

A wealth of evidence has emerged that two related, multisubunit E3 ubiquitin ligase enzymes, the Anaphase Promoting Complex (APC) and the Skp1-Cullin1-F-box complex (SCF), have been considered as the major driving forces governing cell cycle progression [26]. The initial role of Cdc20 was elucidated primarily in regulating cell cycle progression after it was discovered nearly half a century ago [27]. Cells with Cdc20 mutants blocked cell division and stopped cell cycle progression toward anaphase and chromosome segregation. It contains seven WD40 repeats that are necessary for mediating protein-protein interactions [28]. Cdc20 plays an indispensable role during the metaphase to anaphase transition by targeting critical cell cycle regulators including Securin [29] and Cyclin B. It has been also identified that Cdc20 exerts its function through destruction of critical cell cycle regulators (p21) [30] and degradation of conductin governs Wnt/*β*-catenin signaling [31] or Nek2A and Kif18A [32]. Additionally, Cdc20 can cause depletion of endogenous PHF8 that led to prolonged G2 phase and defective mitosis to influence cell cycle [33]. In our study, the top degree hub gene is CDC20 which means that the cell cycle will act as the key role in TAD. The Cytoscape plugin MCODE result also shows that the highest score pathway is relevant with cell cycle. The two analysis methods are consistent and also in accordance with previous studies.

Previous studies also confirm that restricting cell cycle of SMC may lead to disorder of vascular remodeling as an antecedent to the pathological sequelae of CVD [34]. Proliferation of smooth muscle cells (SMCs) is the key event in the pathogenesis of TAD, and VSMCs proliferation is mediated by the cell cycle, under the control of CDC20. Reports show that overexpression of PTEN could regulate the cell cycle in SMCs. The cell cycle is known to be the final convergent point of all proliferative pathways and is the watershed of proliferation [35]. So the abnormal proliferation of VSMCs will be the potentially main cause of pathological vascular remodeling through undermining the vasculature stability and finally lead to vascular disease. Study shows that Talin-1 is significant downregulation of Talin-1 in AD aortic samples and Talin-1 knockdown VSMCs showed increased proliferation with involvement of TAD pathological process. Downregulation of SMAD4 will also increase VSMCs proliferation and cause TAD pathological process [36]. Overexpression of HSP27 inhibition of cell proliferation of VSMCs plays a protective role in TAD [37]. So the cell cycle of VSMCs will play an important role in TAD pathological process. 

In summary, cell cycle may act as a key pathway in TAD pathological process. The relevant gene associated with cell cycle like CDC20 can be used as drug target and diagnostic marker of TAD. However, there are still some limitations: normal aortic tissue used as control group maybe requires more accuracy. Further experimental studies with larger sample size need to confirm cell cycle pathway in TAD.

## Figures and Tables

**Table 1:** The primers of top 10 hub genes.

Gene name	Forward primer	Reverse primer
CDC20	ATGCGCCAGAGGGTTATCAG	AGGATGTCACCAGAGCTTGC
AURKA	GGATATCTCAGTGGCGGACG	GCAATGGAGTGAGACCCTCT
RFC4	GTATCGCGGAAACCTGAGGA	GCATGGTACTTCACCCAGTT
MCM4	CCTTGTCGCGCAGGTACTC	AAGAGGGATAGCTGAAGAATGC
TYMS	CCTTGTCGCGCAGGTACTC	AAGAGGGATAGCTGAAGAATGC
MCM2	ATCGTGGTACTGCTATGGCG	CGGTAGTCCCTTTCCATGCC
DLGAP5	TAAAGCCCCTCCAATCAGCG	GAAGACATCCTGAGCCACCT
FANCI	GTTTGTGGCGGAGTTCTGTG	TCTGCAAATCCCCCGATTCC
BIRC5	GATGACGACCCCATGCAAAG	CGCACTTTCTCCGCAGTTTC
POLE2	GAGAGTGTATCCTGTGCCCG	TCAAAAGCCTTGAAGTTTGCTATC
GAPDH	CGGACCAATACGACCAAATCCG	AGCCACATCGCTCAGACACC

**Table 2:** The significant enriched analysis of differentially expressed genes in thoracic aortic dissection.

Expression	Category	Term	Description	Gene Count	P-Value
DOWN-DEGs	BP	response to unfolded protein	GO:0006986	4	5.03E-03
	BP	positive regulation of glycogen biosynthetic process	GO:0045725	3	6.66E-03
	BP	positive regulation of transcription, DNA-templated	GO:0045893	11	1.06E-02
	BP	vesicle organization	GO:0016050	3	2.23E-02
	BP	sarcomere organization	GO:0045214	3	2.39E-02
	CC	Z disc	GO:0030018	7	3.24E-04
	CC	focal adhesion	GO:0005925	11	9.94E-04
	CC	stress fiber	GO:0001725	4	8.61E-03
	CC	cytoskeleton	GO:0005856	8	2.63E-02
	CC	actin cytoskeleton	GO:0015629	6	2.81E-02
	MF	actin binding	GO:0003779	10	2.70E-04
	MF	structural constituent of muscle	GO:0008307	4	3.94E-03
	MF	phosphatidylserine binding	GO:0001786	3	3.20E-02
	MF	receptor tyrosine kinase binding	GO:0030971	3	4.21E-02
	MF	calcium ion binding	GO:0005509	11	4.61E-02
UP-DEGs	BP	DNA recombination	GO:0006310	5	1.93E-04
	BP	base-excision repair	GO:0006284	5	6.20E-04
	BP	positive regulation of DNA-directed DNA polymerase activity	GO:1900264	3	3.99E-03
	BP	DNA replication initiation	GO:0006270	4	4.91E-03
	BP	DNA repair	GO:0006281	6	5.88E-03
	CC	extracellular exosome	GO:0070062	53	7.40E-04
	CC	spindle microtubule	GO:0005876	5	1.65E-03
	CC	Ctf18 RFC-like complex	GO:0031390	3	3.71E-03
	CC	membrane	GO:0016020	25	4.31E-03
	CC	cytoplasm	GO:0005737	54	2.68E-02
	MF	ATP binding	GO:0005524	35	4.75E-04
	MF	DNA clamp loader activity	GO:0003689	3	7.05E-03
	MF	single-stranded DNA-dependent ATPase activity	GO:0043142	3	8.97E-03
	MF	purine nucleobase binding	GO:0002060	2	3.26E-02
	MF	protein-tyrosine sulfotransferase activity	GO:0008476	2	3.26E-02

## Data Availability

The data used to support the findings of this study are available from the corresponding author upon request.

## Abbreviations

TAD: Thoracic aortic dissection
GEO: The Gene Expression Omnibus
DEGs: The differentially expressed genes
ECM: Extracellular matrix
VSMCs: Vascular smooth muscle cells
MCODE: Molecular Complex Detection
KEGG: Kyoto Encyclopedia of Genes and Genomes
GO: Gene ontology
BP: Biological processes
CC: Cellular component
MF: Molecular function
PPI: Protein-protein interaction
qRT-PCR: Quantitative reverse transcription-PCR
SMCs: Smooth muscle cells.
